# Comparing mortality among adult, general intensive care units in England with varying intensivist cover patterns: a retrospective cohort study

**DOI:** 10.1186/s13054-014-0491-3

**Published:** 2014-08-14

**Authors:** M Elizabeth Wilcox, David A Harrison, Alasdair Short, Max Jonas, Kathryn M Rowan

**Affiliations:** Interdepartmental Division of Critical Care Medicine, Toronto Western Hospital, 399 Bathurst Street, Rm 411-M, 2nd Floor McLaughlin, Toronto, ON M5T2S8 Canada; Intensive Care National Audit & Research Centre (ICNARC), 24 High Holborn, London, WC1V 6AZ UK; Faculty of Intensive Care Medicine (FICM Workforce Advisory Group), Churchill House, 35 Red Lion Square, London, WC1R 4SG UK; Intensive Care, University Hospital, Southampton, SO166YD UK

## Abstract

**Introduction:**

Research has demonstrated that intensivist-led care of the critically ill is associated with reduced intensive care unit (ICU) and hospital mortality. The objective of this study was to evaluate whether a relation exists between intensivist cover pattern (for example, number of days of continuous cover) and patient outcomes among adult general ICUs in England.

**Methods:**

We conducted a retrospective cohort study by using data from a pooled case mix and outcome database of adult general critical care units participating in the Intensive Care National Audit & Research Centre (ICNARC) Case Mix Programme. Consecutive admissions to participating units for the years 2010 to 2011 were linked to a survey of intensivist cover practices. Our primary outcome of interest was mortality at ultimate discharge from acute-care hospital.

**Results:**

The analysis included 80,122 patients admitted to 130 ICUs in 128 hospitals. Multivariable logistic regression analysis was used to assess the relation between intensivist cover patterns (days of continuous cover, grade of physician staffing at nighttime, and frequency of daily handovers) and acute hospital mortality, adjusting for patient case mix. No relation was seen between days of continuous cover by a single intensivist or grade of physician staffing at nighttime and acute hospital mortality. Acute hospital mortality and ICU length of stay were not associated with intensivist characteristics, intensivist full-time equivalents per bed, or years of clinical experience. Intensivist participation in handover was associated with increased mortality (odds ratio, 1.27; 95% confidence interval, 1.04 to 1.55); however, only nine units reported no intensivist participation.

**Conclusions:**

We found no relation between days of continuous cover by a single intensivist or grade of physician staffing at nighttime and patient outcomes in adult, general ICUs in England. Intensivist participation in handover was associated with increased mortality; further research to confirm or refute this finding is required.

**Electronic supplementary material:**

The online version of this article (doi:10.1186/s13054-014-0491-3) contains supplementary material, which is available to authorized users.

## Introduction

For the past two decades, research has demonstrated that input from physicians with special expertise in the care of the critically ill, termed intensivists, improves patient care and outcomes. A recent systematic review of observational studies indicated that comprehensive intensivist-led care, when compared with partial or non-intensivist care, decreased intensive care unit (ICU) and acute hospital mortality, as well as decreasing length of stay (LOS) in both the ICU and the hospital [1]. Based on similar earlier findings [2,3], in 2011, the European Society of Intensive Care Medicine (ESICM) established guidelines for intensivist staffing of ICUs [4], recommending that trained intensivists be the most responsible physicians in the care of critically ill patients and that they should provide, ideally, 24-hour, in-house cover [5].

In a 1999 report by the UK Audit Commission, higher-than-expected acute hospital mortality was reported for ICUs with sessional allocation, where an intensivist worked a set number of sessions each week (for example, every Tuesday morning), compared with those with weekly allocation, in which an intensivist worked in the ICU for a week [6]. This finding was subsequently supported by other observational studies [7,8]. It was hypothesized that weekly cover might improve continuity of care (intensivists are likely to have an improved overall knowledge of a patient’s condition), allow more-timely treatment (more time available to conduct treatment/procedures rather than defer them to the next session), and facilitate communication (less information lost in handovers) [8].

Despite the recommendations of the 1999 Audit Commission report, sessional allocation of staffing for ICUs persists. The UK Intercollegiate Board for Training in Intensive Care Medicine and the Intensive Care Society (ICS) have suggested that the shortages in appropriately and fully trained intensivists may play a role in limiting its implementation [9].

Given the advantages of an intensivist presence, our hypothesis was that greater intensivist exposure within a high-intensity model of care (that is, transfer of care to an intensivist-led team or mandatory consultation of an intensivist) would be associated with a decrease in acute hospital mortality. We examine the relation between intensivist cover pattern (days of continuous cover, grade of physician staffing at nighttime, and frequency of daily handovers) and patient outcomes (risk-adjusted acute hospital mortality and ICU LOS among survivors) in adult, general ICUs in England.

## Methods

### Study design

A prospective survey of ICU intensivist staffing, structures, and care processes was conducted in 2011. The 10-item questionnaire (see Additional file 1) was developed and distributed to 177 clinical leads or clinical directors in 185 adult, general (mixed medical/surgical) ICUs (including combined intensive care/high-dependency units) in England participating in the Intensive Care National Audit & Research Centre (ICNARC) Case Mix Programme, the national clinical audit of adult intensive care. The questionnaire had the following domains: organization, rota, and intensivists.

The questionnaire was developed through a literature review and focus-group consultation with experts in intensive care workforce-related issues. Experts included intensivists, health services researchers, and leaders/representatives from the ICS and Faculty of Intensive Care Medicine (FICM). The questionnaire was further refined by consulting a similar questionnaire developed in the United States (personal communication, Dr. Jeremy Kahn, University of Pittsburgh).

A detailed process of item generation, item reduction, question formatting, and pretesting or piloting of the questionnaire was completed [10]. Before administration, clinical sensibility, validity, and reliability of our questionnaire were assessed in a sample of intensivists. The questionnaire was pilot-tested by eight intensivists, and preliminary data were analyzed to ensure statistical utility (for example, absence of ceiling and floor effects) and clinical sensibility. Some questions were reworded for clarity.

The survey was administered electronically under the auspices of ICNARC, the ICS, the FICM, and the Royal College of Anaesthetists. Consent for participation was voluntary, and implied by the completion and return of the self-administered questionnaire. Any perceived inconsistencies in acquired data were clarified or confirmed by telephone. The London School of Hygiene and Tropical Medicine Ethics Committee approved the study (Study approval number: 010/101).

Unit-level survey responses were linked with patient-level data from the Case Mix Programme database. The database contains raw physiological and diagnostic data needed for the Acute Physiology And Chronic Health Evaluation (APACHE) II and ICNARC risk-prediction models [11,12], in addition to demographics, outcomes (ICU/hospital mortality) and activity (ICU/hospital LOS) data, for consecutive admissions to ICUs participating in the Case Mix Programme. Trained data collectors abstract prospectively recorded clinical data, retrospectively, and the collected data undergo extensive validation both locally and centrally, as detailed previously [13,14]. Data were extracted for all admissions to adult, general ICUs in England between 1 April 2010 and 31 March 2011, corresponding to the timing of survey administration. Readmissions to the ICU within the same acute hospital stay and admissions with missing acute hospital outcomes were excluded from the analysis.

### Statistical analysis

Survey responses were summarized for all responders; only ICUs with complete survey data and Case Mix Programme data were included in the linked analysis. Patient characteristics and outcomes were summarized for admissions to responding and nonresponding units; responding units include both those units included in the linked analysis (complete survey response) and units with an incomplete survey response. Descriptive statistics are presented as number and percentage, mean and standard deviation (SD), or median and interquartile range (IQR), as appropriate.

Analyses of linked, unit- and patient-level data were conducted by using multilevel logistic (for acute hospital mortality) and log-linear (for ICU LOS for survivors) regression models to explore the association between intensivist cover pattern and clinical outcomes, adjusted for the components of the ICNARC risk-prediction model (age, location before admission, CPR within 24 hours before admission, ICNARC Physiology Score and body system of the primary reason for admission) [11], and a random effect of unit.

Structure and intensivist staffing variables included in the multilevel models were as follows: absolute number of rounds per week; absolute number of handovers in 24 hours (averaged over 1 week); intensivist participation in handovers (weekdays only or weekdays and weekends compared with no intensivist participation); maximum number of continuous days of intensivist cover; nighttime cover pattern (intensivist, specialty registrar (dedicated) with intensivist not exclusively on call, specialty registrar (cross cover) with intensivist exclusively on call, specialty registrar (cross cover) with intensivist not exclusively on call, senior or junior medical officer with intensivist exclusively on call, or senior or junior medical officer with intensivist not exclusively on call compared with specialty registrar (dedicated) with intensivist exclusively on call); number of ICU beds; number of full-time equivalent (FTE) intensivists on the ICU staff per bed; and average years of clinical experience of intensivists (weighted by FTE).

Results of the multilevel models are presented as the odds ratio (OR) for acute hospital mortality or the relative effect on ICU LOS (the exponential of the coefficient from the log-linear model) with 95% confidence intervals (CIs). As a sensitivity analysis, the multivariable models were re-run with a binary variable representing multiple compared with single or partial days of intensivist cover in place of the number of continuous days of intensivist cover.

All analyses were conducted with Stata/SE version 10.1.

## Results

**Figure 1:**
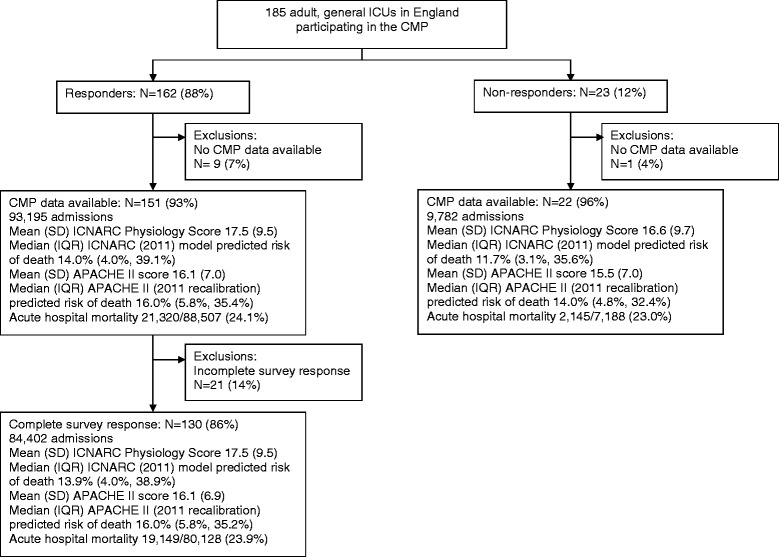
**Numbers of adult general ICUs and patients in the study.**

Survey responses were received from 162 of 185 adult, general ICUs (in 160 of 183 acute hospitals) in England participating in the ICNARC Case Mix Programme (88%; Figure 1). Of these, 151 had sufficient Case Mix Programme data available for inclusion in the analyses, of which 21 units had incomplete survey responses. A cohort of 84,402 admissions to 130 (70%) ICUs (in 128 acute hospitals) comprised the final dataset for analysis.

The ICUs were diverse with respect to the annual number of admissions, number of beds, reported academic status, and geographic region (Strategic Health Authority). ICUs in which the clinical lead responded to the survey were, on average, smaller (in terms of annual admissions and beds) than those that did not (Table 1). Patient characteristics and outcomes were also similar between responding and nonresponding ICUs. Survey responses for all 162 responders and for the 130 ICUs included in the analysis of linked unit- and patient-level data are shown in Table 2. Among responding ICUs, 161 (99%) had a full-time lead or clinical director, and all 162 (100%) reported their unit-cover model as being closed. Four ICUs (3%) reported providing in-house, nighttime intensivist cover (24-hour intensivist cover), although on further investigation, in all cases, this was cross-cover from another service and not dedicated to ICU.

**Table 1:** **Characteristics of the ICUs according to survey response**

	**Responders**				
**Characteristic**	**All (** ***N*** **= 162)**	**Included in analysis (** ***n*** **= 130)**	**Not included in analysis (** ***n*** **= 32)**	**Nonresponders (** ***n*** **= 23)**	***P*** **value**
Annual ICU admissions					0.003
Median	580	640	440	390	
Interquartile range	430-780	440-820	370-680	290-580	
ICU beds, number (%)					0.028
<8	36 (22.2)	29 (22.3)	7 (21.9)	12 (52.2)	
8 to 10	51 (31.5)	32 (24.6)	19 (59.4)	5 (21.7)	
11 to 15	35 (21.6)	33 (25.4)	2 (6.3)	4 (17.4)	
>15	40 (24.7)	36 (27.7)	4 (12.5)	2 (8.7)	
Academic status, number (%)					0.37
University-affiliated hospital	75 (46.3)	59 (45.4)	16 (50.0)	8 (34.8)	
Nonuniversity hospital	87 (53.7)	71 (54.6)	16 (50.0)	15 (65.2)

**Table 2:** **Survey responses from all responders and those ICUs included in the analysis of linked unit-level and patient-level data**

**Characteristic**	**All responders**	**Included in analysis**	***P*** **value**
	**(** ***n*** **= 162)**	**(** ***n*** **= 130)**	
Full-time lead or clinical director, number/total number (%)	161/162 (99.4)	129/130 (99.2)	1.00
Unit format, number (%)			-
Closed	162 (100)	130 (100)	
Open	0 (0)	0 (0)	
Formal rounds in 24 hours (weekday), number (%)			0.97
1	38 (12.5)	30 (23.1)	
2	88 (54.3)	70 (53.8)	
3	30 (18.5)	25 (19.2)	
4 or more	6 (3.7)	5 (3.8)	
Formal rounds in 24 hours (weekend day), number (%)			0.044
0	1 (0.6)	1 (0.8)	
1	83 (51.2)	61 (46.9)	
2	72 (44.4)	64 (49.2)	
3	6 (3.7)	4 (3.1)	
Handover rounds in 24 hours, number (%)			0.26
1	6 (3.7)	3 (2.3)	
2	117 (72.2)	95 (73.1)	
3	37 (22.8)	30 (23.1)	
4	2 (1.2)	2 (1.5)	
Intensivist participation in handover rounds, number (%)			0.56
No intensivist participation	9 (5.6)	6 (4.6)	
Weekdays only	40 (24.8)	33 (25.4)	
Weekdays and weekend days	112 (69.6)	91 (70.0)	
Nighttime cover pattern, number (%)			0.95
Intensivist in-house (dedicated or cross-cover)	4 (2.6)	4 (3.1)	
Specialty registrar (dedicated);			
Intensivist exclusively on-call for the ICU	70 (45.2)	59 (45.4)	
Specialty registrar (dedicated);			
Intensivist not exclusively on-call for the ICU	10 (6.5)	9 (6.9)	
Specialty registrar (cross-cover);			
Intensivist exclusively on-call for the ICU	21 (13.5)	18 (13.8)	
Specialty registrar (cross-cover);			
Intensivist not exclusively on-call for the ICU	30 (19.4)	24 (18.5)	
Senior or junior medical officer;			
Intensivist exclusively on-call for the ICU	17 (11.0)	13 (10.0)	
Senior or junior medical officer;			
Intensivist not exclusively on-call for the ICU	3 (1.9)	3 (2.3)	
Number of intensivists available to staff ICU, number (%)			0.38
<6	24 (15.2)	21 (16.2)	
6-8	71 (44.9)	54 (41.5)	
9-11	35 (22.2)	30 (23.1)	
12+	28 (17.7)	25 (19.2)	
FTE intensivists per bed			0.37
Median	0.50	0.50	
Interquartile range	0.35-0.69	0.35-0.71	
Base specialty of intensivists – no. (%)			0.59
Anesthetics	1,223 (93.4)	1,048 (93.3)	
Medicine	72 (5.5)	61 (5.4)	
Emergency medicine	12 (0.9)	12 (1.1)	
Surgery	2 (0.2)	2 (0.2)	
Years of clinical experience; number (%)			0.88
<3 years	281 (21.5)	238 (21.2)	
4–6 years	249 (19.0)	217 (19.3)	
7–10 years	258 (19.7)	221 (19.7)	
>10 years	521 (39.8)	447 (39.8)	

In the units without in-house, nighttime intensivist cover, the most common nighttime staffing models were a specialty registrar (a trainee in critical care who has finished training in anesthesia, surgery, accident and emergency, or internal medicine; equivalent grade of trainee to a fellow in North America) dedicated to the ICU (80; 52% of ICUs), a specialty registrar cross-covering the ICU in addition to another service (51; 30% of ICUs), and a senior or junior medical officer (a trainee with an equivalent grade of training to a resident in North America), either dedicated or cross-covering (20; 13% of ICUs).

### Unit organization and outcome

In the multilevel, logistic regression model (Table 3), the absolute number of formal rounds per week and the absolute number of handovers in a 24-hour period were not associated with risk-adjusted acute hospital mortality. However, both intensivist participation in handovers during weekdays (Monday through Friday) and during weekdays and weekends were associated with higher risk-adjusted acute hospital mortality compared with no intensivist participation. No significant difference in risk-adjusted acute hospital mortality was found with additional continuous days of intensivist cover. The effect was similar when examining the effect of continuous intensivist cover as multiple compared with single or partial days (OR, 1.11; 95% CI, 0.99 to 1.24).

**Table 3:** **Structure of rounds/handovers, nighttime pattern of physician cover, and intensivist characteristics and acute hospital mortality**

	**Odds ratio (95% CI)**	
	**Univariable**	**Multivariable**
**Structure of rounds/handovers**		
Number of ICU beds (per five additional beds)	0.99	0.98
	(0.95, 1.02)	(0.93, 1.02)
Absolute number of rounds per week (per seven additional rounds)	0.99	1.00
	(0.92, 1.05)	(0.93, 1.06)
Absolute number of handovers in 24 hours (per additional handover)	1.03	1.03
	(0.95, 1.12)	(0.94, 1.12)
Intensivist participation in handover rounds (versus no intensivist participation)		
Weekdays only	1.29	1.33
	(1.04, 1.59)	(1.07, 1.65)
Weekdays and weekends	1.24	1.27
	(1.02, 1.52)	(1.04, 1.56)
Maximum continuous days intensivist cover (per additional day)	1.01	1.02
	(0.99, 1.03)	(1.00, 1.04)
**Nighttime pattern of physician cover**		
Nighttime cover pattern (versus specialty registrar (dedicated); intensivist exclusively on call)		
Intensivist	0.98	0.96
	(0.77, 1.26)	(0.76, 1.21)
Specialty registrar (dedicated); intensivist not exclusively on call	0.98	0.91
	(0.82, 1.16)	(0.75, 1.10)
Specialty registrar (cross-cover); intensivist exclusively on call	1.05	1.02
	(0.92, 1.19)	(0.90, 1.16)
Specialty registrar (cross-cover); intensivist not exclusively on call	1.03	1.01
	(0.91, 1.15)	(0.88, 1.17)
Senior or junior medical officer; Intensivist exclusively on call	1.01	0.99
	(0.88, 1.17)	(0.86, 1.15)
Senior or junior medical officer; intensivist not exclusively on call	1.16	1.05
	(0.88, 1.54)	(0.78, 1.41)
**Intensivist characteristics**		
Full-time equivalent (FTE) intensivists per bed (per additional 0.25 FTE)	1.00	0.99
	(0.97, 1.02)	(0.96, 1.03)
Clinical experience of intensivists weighted by FTE (per additional year)	0.98	0.98
	(0.96, 1.01)	(0.96, 1.01)
**Patient-level variables** ^a^		
Age (per 10 years)	-	1.39
		(1.37, 1.41)
Location before admission (versus Emergency Department)		
Clinic or home	-	0.97
		(0.81, 1.16)
Other critical care unit	-	1.27
		(1.16, 1.38)
Theater (elective/scheduled surgery)	-	0.29
		(0.23, 0.35)
Theater (emergency/urgent surgery)	-	0.62
		(0.51, 0.75)
Ward or intermediate-care area	-	1.60
		(1.52, 1.69)
CPR within 24 hours before dmission	-	2.55
		(2.35, 2.77)
ICNARC Physiology Score (per 5 points)	-	1.88
		(1.86, 1.91)
Primary reason for admission (versus Respiratory)		
Cardiovascular (nonsurgical)	-	0.85
		(0.79, 0.92)
Cardiovascular (surgical)	-	1.20
		(0.98, 1.47)
Dermatologic (nonsurgical)	-	0.92
		(0.73, 1.16)
Dermatologic (surgical)	-	1.03
		(0.70, 1.50)
Endocrine (nonsurgical)	-	0.45
		(0.40, 0.50)
Endocrine (surgical)	-	0.84
		(0.56, 1.27)
Gastrointestinal (nonsurgical)	-	0.98
		(0.90, 1.06)
Gastrointestinal (surgical)	-	1.27
		(1.05, 1.53)
Genitourinary (nonsurgical)	-	0.52
		(0.48, 0.57)
Genitourinary (surgical)	-	0.73
		(0.56, 0.94)
Hematologic/immunologic (nonsurgical)	-	1.53
		(0.28, 1.82)
Hematologic/immunologic (surgical)	-	1.87
		(0.84, 4.19)
Musculoskeletal (nonsurgical)	-	0.88
		(0.72, 1.07)
Musculoskeletal (surgical)	-	1.13
		(0.89, 1.44)
Neurologic (nonsurgical)	-	1.78
		(1.65, 1.92)
Neurologic (surgical)	-	2.34
		(1.86, 2.94)

^a^Each univariable model was adjusted for the same patient-level variables (odds ratios not shown).

Increasing ICU size was associated with a 7% increase in ICU LOS among survivors for each additional five beds (Table 4). In univariable models (adjusted for patient-level variables only), absolute number of rounds per week was associated with an increase in ICU LOS, and number of handovers in 24 hours was associated with a decrease in ICU LOS, but these effects did not persist when adjusted for other unit-level variables. Participation of intensivists in handover and additional days of intensivist cover were not associated with any difference in ICU LOS. For multiple as compared with single or partial days of intensivist cover, the relative effect was 0.99 (95% CI, 0.91 to 1.07).

**Table 4:** **Structure of rounds/handovers, nighttime pattern of physician cover, and intensivist characteristics and ICU length of stay among survivors**

	**Relative effect (95% CI)**	
	**Univariable**	**Multivariable**
**Structure of rounds/handovers**		
Number of ICU beds (per five additional beds)	1.08	1.07
	(1.06, 1.11)	(1.03, 1.10)
Absolute number of rounds per week (per additional seven rounds)	1.06	1.04
	(1.02, 1.12)	(0.98, 1.09)
Absolute number of handovers in 24 hours (per additional handover)	0.93	0.95
	(0.87, 0.99)	(0.89, 1.01)
Intensivist participation in handover rounds (versus no intensivist participation)		
Weekdays only	1.03	1.00
	(0.88, 1.20)	(0.85, 1.17)
Weekdays and weekends	1.06	0.99
	(0.91, 1.23)	(0.85, 1.15)
Maximum continuous days intensivist cover (per additional day)	1.00	1.00
	(0.99, 1.02)	(0.98, 1.01)
**Nighttime pattern of physician cover**		
Nighttime cover pattern (versus specialty registrar (dedicated); intensivist exclusively on call)		
Intensivist	0.91	0.93
	(0.76, 1.09)	(0.78, 1.12)
Specialty registrar (dedicated); intensivist not exclusively on call	0.92	1.00
	(0.81, 1.04)	(0.87, 1.15)
Specialty registrar (cross-cover); intensivist exclusively on call	0.97	1.00
	(0.88, 1.07)	(0.90, 1.10)
Specialty registrar (cross-cover); intensivist not exclusively on call	0.91	0.97
	(0.84, 1.00)	(0.87, 1.07)
Senior or junior medical officer; intensivist exclusively on call	1.05	1.05
	(0.94, 1.17)	(0.94, 1.17)
Senior or junior medical officer; intensivist not exclusively on call	0.89	1.01
	(0.72, 1.10)	(0.81, 1.26)
**Intensivist characteristics**		
Full-time equivalent (FTE) intensivists per bed (per additional 0.25 FTE)	0.97	0.98
	(0.95, 0.99)	(0.96, 1.00)
Clinical experience of intensivists weighted by FTE (per additional year)	0.99	0.99
	(0.97, 1.01)	(0.97, 1.01)
Patient-level variables*		
Age (per 10 years)	-	1.03
		(1.02, 1.03)
Location before admission (versus Emergency Department)		
Clinic or home	-	0.90
		(0.83, 0.96)
Other critical care unit	-	1.80
		(1.74, 1.87)
Theatre (elective/scheduled surgery)	-	0.61
		(0.58, 0.64)
Theatre (emergency/urgent surgery)	-	0.75
		(0.71, 0.79)
Ward or intermediate care area	-	1.19
		(1.17, 1.22)
CPR within 24 hours before admission	-	1.02
		(0.98, 1.07)
ICNARC Physiology Score (per 5 points)	-	1.37
		(1.36, 1.38)
Primary reason for admission (versus Respiratory)		
Cardiovascular (nonsurgical)	-	0.71
		(0.69, 0.73)
Cardiovascular (surgical)	-	1.11
		(1.05, 1.16)
Dermatologic (nonsurgical)	-	0.86
		(0.78, 0.95)
Dermatologic (surgical)	-	1.12
		(1.01, 1.24)
Endocrine (nonsurgical)	-	0.61
		(0.59, 0.63)
Endocrine (surgical)	-	0.95
		(0.88, 1.03)
Gastrointestinal (nonsurgical)	-	0.88
		(0.85, 0.91)
Gastrointestinal (surgical)	-	1.20
		(1.15, 1.26)
Genitourinary (nonsurgical)	-	0.64
		(0.62, 0.66)
Genitourinary (surgical)	-	0.95
		(0.90, 1.01)
Hematologic/immunologic (nonsurgical)	-	0.82
		(0.76, 0.89)
Hematologic/immunologic (surgical)	-	1.22
		(0.92, 1.61)
Musculoskeletal (nonsurgical)	-	1.01
		(0.95, 1.09)
Musculoskeletal (surgical)	-	0.92
		(0.87, 0.97)
Neurologic (nonsurgical)	-	0.71
		(0.69, 0.73)
Neurologic (surgical)	-	0.15
		(1.09, 1.22)

*Each univariable model was adjusted for the same patient-level variables (relative effects not shown).

### Nighttime cover pattern and outcome

In the multilevel logistic regression model, nighttime cover pattern was not associated with risk-adjusted acute hospital mortality (Table 3). Further, no significant difference in ICU LOS for survivors was seen with any patterns in nighttime cover, as compared with the most common pattern of a dedicated specialty registrar and an intensivist exclusively on call to the ICU (Table 4).

### Clinical care concentration/experience of intensivists and outcome

No significant difference was seen in risk-adjusted acute hospital mortality with increasing intensivist staffing, as measured by the FTE per bed among all consultants on the ICU staff (Table 3). In the univariable analysis, for the addition of each 0.25 intensivist FTE per bed, ICU LOS for survivors decreased by 3%, but this effect was not significant after adjustment for other unit-level variables (Table 4). The average experience of the intensivist within each ICU was also not associated with risk-adjusted acute hospital mortality (Table 3) or ICU LOS for survivors (Table 4).

## Discussion

This study failed to demonstrate a relation between days of continuous cover by a single intensivist or grade of physician staffing at nighttime and risk-adjusted acute hospital mortality in patients admitted to adult, general ICUs in England. Further, risk-adjusted acute hospital mortality and ICU LOS were not associated with characteristics specific to the intensivist, such as intensivist FTE per bed or years of clinical experience. Unexpectedly, intensivist participation in handover was, however, associated with increased risk-adjusted acute hospital mortality.

Previous studies have shown decreased ICU and hospital mortality and LOS in closed units with transfer of primary care responsibility to a single intensivist-led team or mandatory intensivist consultation [1-3]. This question, however, is relevant only in the United States, where most ICUs are open [15-17]. In our study, all of the units were closed, which limited our ability to demonstrate differences in outcome for this covariate. Likewise, the format of sessional as compared with weekly cover by a single intensivist is most consistent with a European staffing pattern. In contrast to our results, the 1999 UK Audit Commission found higher acute hospital mortality in units with sessional allocation compared with weekly cover. This finding pre-dates widespread implementation of standard ICU processes of care, such as ventilator-care bundles and lung-protective ventilation protocols, which may mitigate the effect of increased handovers that are inevitably required with sessional cover [6].

Investigations to date have focused on patient-related outcomes; as a result, little is known about the consequences of work schedules in intensive care on intensivists themselves. In a cluster randomized study of 2 weeks of continuous cover interrupted by weekend cover by a cross-covering physician, compared with 14 days of continuous cover by a single consultant, the intervention group reported reduced burnout, improved work-life balance, and decreased work distress, without influencing patient outcomes [18]. Our data also suggest that less-cumbersome patterns of intensivist cover might be feasible. Sessional cover, which was not associated with increased risk-adjusted acute hospital mortality or ICU LOS, could reduce burnout, as it allows a limited number of hours or days of ICU cover.

In our study, intensivist participation in handover was associated with increased risk-adjusted acute hospital mortality. This is likely to be a chance finding, as only nine of 130 units reported no intensivist participation, or it may be confounded by an unknown characteristic in those nine units.

However, alternate possibilities are that intensivist participation during handover distracts from other patient-care tasks that have a direct positive impact on patient outcome, or that intensivist participation negatively influences the handover process. A potential mechanism for the latter effect is communication of an “authoritative” summary of each patient’s clinical status that is insufficiently challenged by the on-call team when patients deteriorate. This hypothesis is supported by a single-center study that found more nighttime decisions and lower ICU mortality in patients exposed to on-call cross-covering fellows [19]. These findings contradict the dominant hypothesis that cross-coverage is associated with worse outcomes, and suggest that a “second look” by cross-covering fellows may mitigate cognitive errors.

Further research is needed to determine the role of an intensivist in handover and possibly to improve approaches to handover communication.

In a recent US study, nighttime intensivist staffing was associated with a reduction in risk-adjusted hospital mortality in ICUs with low-intensity daytime staffing [20]. However, among ICUs with high-intensity daytime staffing, nighttime intensivist staffing conferred no benefit. A second, single-center, randomized controlled trial comparing 7 days of nighttime staffing with in-hospital intensivists with daytime intensivists who were available by pager overnight showed no differences in patient outcomes [21]. In our study, greater than 95% of units were high intensity by US definitions, and our finding that the level of experience of the nighttime physician on-call did not influence patient outcomes is consistent with previous work. However, our results should be interpreted with caution, given that very few (*n* = 4 of 130; 2.6%) had 24-hour in-house (dedicated or cross-covering) intensivist staffing.

The characteristics of the intensivists providing clinical care were hypothesized to be a confounder of the relation between cover pattern and risk-adjusted acute hospital mortality. It would make intuitive sense that a intensivist with more experience or who spent the majority of the time at the bedside doing clinical work would make up for other shortcomings in cover patterns. We found little evidence, however, to support this hypothesis, and, as a result, it is likely that unit-level factors may be more responsible for patient outcomes (for example, volume-outcome relation [22,23]; nurse-to-patient ratio [24,25] or other aspects of nursing organization; implementation of best practices) [15,26].

Examination of international differences in the provision of care to critically ill patients may provide further insight into unit-level characteristics responsible for stable mortality rates across different intensivist cover patterns [27].

Strengths of our study include a rigorous approach to the development and testing of our questionnaire, a high response rate (limiting the risk of response bias), adjustment for severity of illness with a well-validated dataset, and a comprehensive set of covariates included in the analysis.

Limitations of this study include small variations in certain variables of interest, which limits the power to detect very modest differences in outcomes across some aspects of cover patterns. Although we adjusted for variables related to patient case mix and organizational issues at the hospital and ICU levels, the multivariable analysis would not take into account other unmeasured factors, such as adherence to evidence-based medicine guidelines or nurse-to-patient ratios. Further, our ability to detect a relation between intensivist cover pattern and outcome may also have been reduced by nondifferential measurement bias, in which all variables (whether exposure or covariate) have the same error rate or the same probability for misclassification [28,29], because of random errors in survey responses, a phenomenon that would bias associations to the null. However, we attempted to minimize this possibility by developing the survey with standard rigorous methods and piloting extensively before administration to ensure a common interpretation of questions.

Future research should potentially focus on other elements of service delivery and organization. Comprehensive staffing models, not limited to intensivist cover but evaluating all staff involved, may be one approach. Another approach may be to evaluate other factors, such as adherence to evidence-based practices or processes (for example, lung-protective ventilation strategies). If these elements of service delivery and organization are evaluated as effective and can be better understood, then other units may be able to implement similar models of care.

## Conclusions

No relation was seen between days of continuous cover by a single intensivist, grade of physician staffing at nighttime, or intensivist characteristics (for example, intensivist full-time equivalents per bed) and risk-adjusted acute hospital mortality.

## Key messages

- No relation was seen between days of continuous cover by a single intensivist or grade of physician staffing at nighttime and acute risk-adjusted hospital mortality.
- Acute risk-adjusted hospital mortality and ICU length of stay were not associated with intensivist characteristics (for example, intensivist full-time equivalents per bed or years of clinical experience).

### Ethical approval

London School of Hygiene and Tropical Medicine Ethics Committee approval number: 010/101.

## Additional file

Additional file 1:
**Consultant Cover Questionnaire.**

## Abbreviations

APACHE: Acute physiology and chronic health evaluation
CI: confidence interval
ESICM: European Society of Intensive Care Medicine
FICM: Faculty of Intensive Care Medicine
FTE: full-time equivalent
ICNARC: Intensive Care National Audit & Research Centre
ICS: Intensive Care Society
ICU: intensive care unit
IQR: interquartile range
LOS: length of stay
OR: odds ratio
SD: standard deviation

## References

[1] Wilcox ME, Chong CAKY, Niven DJ, Rubenfeld GD, Rowan KM, Wunsch H, Fan E (2013). Do intensivist staffing patterns influence hospital mortality following ICU admission? A systematic review and meta-analyses. Crit Care Med.

[2] Pronovost PJ, Angus DC, Dorman T, Robinson KA, Dremsizov TT, Young TL (2002). Physician staffing patterns and clinical outcomes in critically III patients: a systematic review. JAMA.

[3] Young MP, Birkmeyer JD (2000). Potential reduction in mortality rates using an intensivist model to manage intensive care units. Effect Clin Pac.

[4] Valentin A, Ferdinande P, Improvement EWGoQ (2011). Recommendations on basic requirements for intensive care units: structural and organizational aspects. Intensive Care Med.

[5] Kahn JM, Brake H, Steinberg KP (2007). Intensivist physician staffing and the process of care in academic medical centres. Qual Saf Health Care.

[6] (1999). The place of efficient and effective critical care services within the acute hospital.

[7] Royle P, Bramall J, Norrington A (2008). The effect of introducing a consultant weekly working pattern on mortality in a critical care unit. J Intensive Care Med.

[8] Kalman S, Szakmany T (2010). Consultant working patterns could impact significantly on ICU length of stay: evaluation of daily vs weekly cover. Intensive Care Medicine Conference: 23rd Annual Congress of the European Society of Intensive Care Medicine, ESICM Barcelona Spain Conference Start.

[9] Gunning K, Gillbe C, on behalf of the Council of Intensive Care Society and the Intercollegiate Board of Training in Intensive Care Medicine: **Standards for Consultant Staffing of Intensive Care Units.** *ᅟ* 2006. Available; http://www.ics.ac.uk.

[10] Burns KEA, Duffett M, Kho ME, Meade MO, Adhikari NKJ, Sinuff T, Cook DJ, Group A (2008). A guide for the design and conduct of self-administered surveys of clinicians. CMAJ.

[11] Harrison DA, Parry GJ, Carpenter JR, Short A, Rowan K (2007). A new risk prediction model for critical care: the Intensive Care National Audit & Research Centre (ICNARC) model. Crit Care Med.

[12] Knaus WA, Draper EA, Wagner DP, Zimmerman JE (1985). APACHE II: a severity of disease classification system. Crit Care Med.

[13] Harrison DA, Brady AR, Rowan K (2004). Case mix, outcome and length of stay for admissions to adult, general critical care units in England, Wales and Northern Ireland: the Intensive Care National Audit & Research Centre Case Mix Programme Database. Crit Care (London).

[14] The Information Centre. List of databases: **The Information Centre. List of databases.** *ᅟ* 2010. Available: http://www.icapp.nhs.uk.

[15] Lilly CM, Zuckerman IH, Badawi O, Riker RR (2011). Benchmark data from more than 240,000 adults that reflect the current practice of critical care in the United States. Chest.

[16] Angus DC, Shorr AF, White A, Dremsizov TT, Schmitz RJ, Kelley MA, on behalf of the Committee on Manpower for Pulmonary and Critical Care Societies (COMPACCS) (2006). Critical care delivery in the United States: distribution of services and compliance with Leapfrog recommendations. Crit Care Med.

[17] Angus DC, Kelley MA, Schmitz RJ, White A, Popovich J (2000). Current and projected workforce requirements for care of the critically ill and patients with pulmonary disease. JAMA.

[18] Ali NA, Hammersley J, Hoffmann SP, O'Brien JM, Phillips GS, Rashkin M, Warren E, Garland A, Midwest Critical Care C (2011). Continuity of care in intensive care units: a cluster-randomized trial of intensivist staffing. Am J Respir Crit Care Med.

[19] Amaral ACK-B, Barros BS, Barros CCPP, Innes C, Pinto R, Rubenfeld GD (2014). nighttime cross-coverage is associated with decreased intensive care unit mortality: a single-center study. Am J Respir Crit Care Med.

[20] Wallace DJ, Angus DC, Barnato AE, Kramer AA, Kahn JM (2012). Nighttime intensivist staffing and mortality among critically ill patients. N Engl J Med.

[21] Kerlin MP, Small DS, Cooney E, Fuchs BD, Bellini LM, Mikkelsen ME, Schweickert WD, Bakhru RN, Gabler NB, Harhay MO, Kerlin MP, Small DS, Cooney E, Fuchs BD, Bellini LM, Mikkelsen ME, Schweickert WD, Bakhru RN, Gabler NB, Harhay MO, Hansen-Flaschen J, Halperin SD (2013). A randomized trial of nighttime physician staffing in an intensive care unit. N Engl J Med.

[22] Kahn JM, Goss CH, Heagerty PJ, Kramer AA, O'Brien CR, Rubenfeld GD (2006). Hospital volume and the outcomes of mechanical ventilation. N Engl J Med.

[23] Shahin J, Harrison DA, Rowan KM (2012). Relation between volume and outcome for patients with severe sepsis in United Kingdom: retrospective cohort study. BMJ.

[24] Penoyer DA (2010). Nurse staffing and patient outcomes in critical care: a concise review. Crit Care Med.

[25] Amaravadi RK, Dimick JB, Pronovost PJ, Lipsett PA (2000). ICU nurse-to-patient ratio is associated with complications and resource use after esophagectomy. Intensive Care Med.

[26] Hewson-Conroy KM, Burrell AR, Elliott D, Webb SAR, Seppelt IM, Taylor C, Glass P (2011). Compliance with processes of care in intensive care units in Australia and New Zealand: a point prevalence study. Anaesth Intensive Care.

[27] Wunsch H, Angus DC, Harrison DA, Linde-Zwirble WT, Rowan KM (2011). Comparison of medical admissions to intensive care units in the United States and United Kingdom. Am J Respir Crit Care Med.

[28] Hsieh C-C (1991). The effect of non-differential outcome misclassification on estimates of the attributable and prevented fraction. Stat Med.

[29] Blakely T, McKenzie S, Carter K (2013). Misclassification of the mediator matters when estimating indirect effects. J Epidemiol Community Health.

