# Supplementary material for: Comparing mortality among adult, general intensive care units in England with varying intensivist cover patterns: a retrospective cohort study
Source: Crit Care. 2014 Aug 14;18(4):491. doi: 10.1186/s13054-014-0491-3 (PMC4159542; doi:10.1186/s13054-014-0491-3)
Supplement: Additional file 1: — Consultant Cover Questionnaire. [file 13054_2014_491_MOESM1_ESM.pdf]

Additional file 1: Consultant Cover Questionnaire

icnarc | intensive care national audit & research centre

Tavistock House
Tavistock Square
London WC1H 9HR
tel +44 (0)20 7388 2856
fax +44 (0)20 7388 3759
email icnarc@icnarc.org

UK Consultant Cover Census (UK-3Cs)

Association between consultant cover and patient outcome in adult general critical care units

This survey has been designed to elicit the information required about consultant cover to explore the relationship between the extent and organisation of consultant cover and its impact on patient outcome in UK adult general critical care units.

The results will be presented at the ICS State of the Art Meeting 2011 and shared with the FICM, ICS and Royal College of Anaesthetists to avoid repetition.

Organisation

The first set of questions ask about how your critical care unit is organised and managed.

1a	Are you the Lead or Clinical Director for your unit?	Yes ☐ No ☐
1b	If not, does your unit have a Lead or Clinical Director?	Yes ☐ No ☐
2	Would you describe your unit as being an open unit?<br>*(By open, we mean a unit where any physician can admit patients to your unit and consultation from an intensive care consultant is at the discretion of that physician - as compared with a closed unit where an intensive care consultant oversees all admission and discharge decisions)*	Yes ☐ No ☐
3	On average, how many times per day do formal rounds occur in your unit on a weekday (Monday to Friday)?<br>*(By formal rounds, we mean multi-disciplinary rounds with intensive care consultants, trainees, bedside nurses, and/or other team members such as pharmacy)*	
4	On average, how many times per day do formal rounds occur in your unit on a weekend day (Saturday to Sunday)?	

Rota

The second set of questions ask about how care and the rota are arranged for your critical care unit.

5	Describe the pattern of consultant cover (or rota) for your unit (alternatively, you can attach an electronic copy of your unit rota).<br>*Please provide the following details in your free-text description:*

Registered as a company limited by Guarantee • Registered No. 2946727 (England) • Registered Charity No. 1039417

	(1) whether consultants work a partial shift or full shift system;<br>*(2) if they work a three day or a split weekend;*<br>*(3) state the maximum number of consecutive days on-call;*<br>*(4) whether there is either single day or continuous days cover of the unit and, if continuous days, then please state how many; and*<br>*(5) the number of consultants on the on-call rota for your unit (e.g. 1 in 4).*<br><br>[free-text]
6	Are your consultants responsible exclusively for the on-call for your unit? Yes ☐ No ☐<br><br>*(By exclusively, we mean not responsible for other areas such as obstetrics, theatres, etc.)*
7a<br><br>7b	What is the highest seniority of person on duty and available **IN** the hospital overnight? (i.e. available at the bedside within five minutes)<br><br>Do you have a non-consultant, second tier, on-call for your unit overnight? (i.e. possibly, at times, available only by bleep) Yes ☐ No ☐<br><br>*(By overnight, we mean for the length of the night after evening handover has occurred)*
8a<br><br>8b<br><br>8c	How many times do handover rounds occur in your unit in a 24-hour period?<br><br>On average, does one or more consultants participate in handover rounds on a weekday (Monday-Friday)? Yes ☐ No ☐<br><br>On average, does one or more consultants participate in handover rounds on a weekend day (Saturday-Sunday)? Yes ☐ No ☐
9a<br><br>9b	Has the consultant cover model changed for your unit in the past five years? Yes ☐ No ☐<br><br>If yes, how? [free-text]

Consultants

The third and last set of questions asks about the characteristics of consultants working in your critical care unit.

10. Please provide a response to every section for each consultant working in your unit.

	Consultant 1	Consultant 2	Consultant 3	Consultant 4	Consultant 5	Consultant 6	Consultant 7	Consultant 8	Consultant 9
Number of years as a consultant									
Parent specialty									
Number of contracted PAs (fixed/scheduled)									
Number of contract DCC for ICM									
Intensive care qualifications									

	Consultant 10	Consultant 11	Consultant 12	Consultant 13	Consultant 14	Consultant 15	Consultant 16	Consultant 17	Consultant 18
Number of years as a consultant									
Parent specialty									
Number of contracted PAs (fixed/scheduled)									
Number of contracted DCC for ICM									
Intensive care qualifications									

(PAs = Programmed Activities; DCCs = Direct Clinical Care; ICM = Intensive Care Medicine)

Thank you for taking the time to complete this questionnaire.
If you have any queries, please email to XX@icnarc.org.
